# A novel hybrid model for species distribution prediction using neural networks and Grey Wolf Optimizer algorithm

**DOI:** 10.1038/s41598-024-62285-8

**Published:** 2024-05-20

**Authors:** Hao-Tian Zhang, Ting-Ting Yang, Wen-Ting Wang

**Affiliations:** School of Mathematics and Computer Science, Northwest Minzu University, Lanzhou, 730030 People’s Republic of China

**Keywords:** Ecological modelling, Ecological modelling

## Abstract

Neural networks are frequently employed to model species distribution through backpropagation methods, known as backpropagation neural networks (BPNN). However, the complex structure of BPNN introduces parameter settings challenges, such as the determination of connection weights, which can affect the accuracy of model simulation. In this paper, we integrated the Grey Wolf Optimizer (GWO) algorithm, renowned for its excellent global search capacity and rapid convergence, to enhance the performance of BPNN. Then we obtained a novel hybrid algorithm, the Grey Wolf Optimizer algorithm optimized backpropagation neural networks algorithm (GNNA), designed for predicting species’ potential distribution. We also compared the GNNA with four prevalent species distribution models (SDMs), namely the generalized boosting model (GBM), generalized linear model (GLM), maximum entropy (MaxEnt), and random forest (RF). These models were evaluated using three evaluation metrics: the area under the receiver operating characteristic curve, Cohen’s kappa, and the true skill statistic, across 23 varied species. Additionally, we examined the predictive accuracy concerning spatial distribution. The results showed that the predictive performance of GNNA was significantly improved compared to BPNN, was significantly better than that of GLM and GBM, and was even comparable to that of MaxEnt and RF in predicting species distributions with small sample sizes. Furthermore, the GNNA demonstrates exceptional powers in forecasting the potential non-native distribution of invasive plant species.

## Introduction

Species distribution models (SDMs) use known geographical occurrences of species and corresponding environmental conditions, such as bioclimatic variables and abiotic variables, to predict the potential distribution of species^1–3^. SDMs have become important tools for ecologists to study ecological issues such as species diversity^4–6^, species conservation^7–9^ and biological invasions^10,11^. In the last decades, a large number of SDMs have been proposed, including regression models (e.g., generalized linear model, GLM)^12–15^, classification models (e.g., generalized boosting model, GBM)^16–18^, complex models (e.g., random forest, RF; maximum entropy, MaxEnt)^16,19–21^, and ensemble models^22,23^. Notably, SDMs such as GLM, GBM, MaxEnt, and RF, are extensively applied in investigating ecological and evolutionary theories^24,25^, assessing climate change impacts^8,26,27^, managing invasive species^10,11^, and identifying conservation areas^7,8^.

Despite their widespread use, the predictive performance of SDMs can varies significantly across different algorithms^2,3,28,29^, posing challenges for reliable forecasts^30–32^. Most research in this filed has focused on comparing the predictive success of various SDMs, endorsing those with superior performance^2,3,33–35^. However, there are few studies on optimization of SDMs that are abandoned due to poor predictive performance^36^. With the development of machine learning, backpropagation neural networks (BPNN) have gained advantages in ecological research where data rarely meet parametric statistical assumptions and non-linear relationships are prevalent^37–39^. However, BPNN also have some disadvantages, such as high dependency on the initial weights, the tendency to be trapped in the local optimum, and slow convergence^38,40,41^, which are particularly pronounced in species distribution predictions^3,28^.

Swarm intelligence optimization algorithms (SIOAs), known for their simplicity, flexibility, and high efficiency, have been used as the primary technique to solve global optimization problems^42–44^. It should be mentioned that the SIOAs mainly introduce randomness in the search process to reduce the possibility of falling into the local optimum^42^. Therefore, it is of practical significance to use the SIOAs to obtain the optimal solution to the global optimization problem. In the past decades, the SIOAs has developed rapidly and becomes a hotspot in many fields^42–48^. So far, many different types of SIOAs have been proposed, such as the Grey Wolf Optimizer (GWO) algorithm^43^, the butterfly optimization algorithm (BOA)^44^, and the sparrow search algorithm (SSA)^42^, each demonstrating success across different optimization tasks^41,49^.

Motivated by these developments, our study introduced a novel hybrid algorithm that leverages the GWO to enhance the BPNN’s predictive performance for species distribution. We detailed the construction of this hybrid algorithm and evaluated its performance against BPNN and the prevalent SDMs (GBM, GLM, MaxEnt, and RF) using data on 23 species. Additionally, we explored the hybrid model’s ability to predict the spatial distribution of an invasive species, aiming to showcase its effectiveness in spatial distribution prediction.

## Materials and methods

### Backpropagation neural networks and Grey Wolf Optimizer algorithm

Backpropagation neural networks (BPNN) are capable of handling both continuous and categorical data^40,50^. They exhibit some attractive properties, including the ability to capture nonlinearity and tolerance noise, but they also have some drawbacks, such as being highly dependent on initial solutions and falling into the local optimum^38,40,41^. The Grey Wolf Optimizer (GWO) algorithm can effectively balance local optimization and global search with its adaptive convergence factor and information feedback mechanism and obtain high convergence speed and solution accuracy^43^.

### Construction of the hybrid algorithm

In this paper, we proposed a novel hybrid algorithm for predicting the potential distribution of species, called Grey Wolf Optimizer algorithm optimized backpropagation neural networks algorithm (GNNA). Specifically, we used the BPNN to construct GNNA. GNNA is not a simple combination of GWO and BPNN but uses the good global search ability and fast convergence ability of GWO to determine the optimal threshold and optimal weight of BPNN. The specific GNNA process is as follows:

1. Determine the basic structure of the BPNN. The three-layer BPNN was selected, the number of nodes in the hidden layer was determined to be 5 and the training set and test set were randomly generated according to 4:1.
2. Initialize the basic parameters. The gray wolf population size was set as 20, the maximum number of iterations was 100, the upper bound of the gray wolf was 1, and the lower bound of the gray wolf was − 1. Initialize the gray wolf position and parameters *A*, *a* and *C*. The dimension of each gray wolf position information was calculated according to the number of layers in each layer of BPNN (dimension = input layer number × hidden layer number + hidden layer number + hidden layer number × output layer number + output layer number).
3. Determine the fitness function. The activation function in the hidden layer and the output layer were adopted Sigmoid type function. The learning rate was 0.01 and the training goal was 0.00001.
4. Calculate the fitness values of all search agents according to the threshold and weight and update the position information of the remaining gray wolves $\omega$ and parameters *A*_*i*_, *a* and *C*_*i*_.
5. Divide the data into test data and training data, and record the optimal search agent and its corresponding error.
6. Determine whether the maximum number of iterations was met. If the condition was met, terminate the cycle; otherwise, repeat steps (4) to (6).
7. Get the result. The final position of the gray wolf $\alpha$, the minimum error of the position of the gray wolf $\alpha$, and error between test data and training data.

Update the gray wolf position according to the following equations. First, calculate the distance vectors between the individual and the prey (Eqs. 1 and 2).

1 $$C_{i} \left( t \right) = 2r_{i} \left( t \right)\left( {i{ = 1,2,3}} \right)$$

2 $$D_{p} \left( t \right) = \left| {C_{i} \left( t \right) \circ X_{p} \left( t \right) - X(t)} \right|\left( {i = 1,2,3; p = \alpha ,\beta ,\delta } \right)$$

where, $C_{i} \left( t \right)\left( {i = 1,2,3} \right)$ represents the random vectors; $r_{i} \left( t \right)\left( {i = 1,2,3} \right)$ represents the random vectors in which every element is in [0,1]; $D_{p} \left( t \right)(p = \alpha ,\beta ,\delta )$ represents the distance vectors between *p* and other individuals, $\circ$ represents the Hadamard product, || represents the absolute value of each element in the vectors; $X_{p} \left( t \right)(p = \alpha ,\beta ,\delta )$ represents the current position of *p*; $X(t)$ represents the current position of the gray wolf.

Second, the positions of the first three wolves are updated according to the following equations:

3 $$A_{i} \left( t \right) = 2a\left( t \right) \circ r_{i + 3} \left( t \right) - a\left( t \right)\left( {i = 1,2,3} \right)$$

4 $$X_{i} \left( t \right) = X_{p} \left( t \right) - A_{i} \left( t \right) \circ D_{p} (t)\left( {i = 1,2,3; p = \alpha ,\beta ,\delta } \right)$$

where, $A_{i} \left( t \right)(i = 1,2,3)$ represents the convergence vector; $r_{i + 3} \left( t \right)(i = 1,2,3)$ represents the random vectors in which every element is in [0,1]; components of $a(t)$ are linearly decreased from 2 to 0 during iteration; $X_{i} \left( t \right)\left( {i = 1,2,3} \right)$ represents the updated position of the first three wolves.

Finally, adjust the position of the offspring gray wolf according to the following equations:

5 $$\omega_{i} = \frac{{\left\| {X_{i} \left( t \right)} \right\|}}{{\mathop \sum \nolimits_{j = 1}^{3} \left\| {X_{j} \left( t \right)} \right\|}}\left( {i = 1,2,3} \right)$$

6 $$X_{\omega } (t + 1) = \frac{{\omega_{1} X_{1} \left( t \right) + \omega_{2} X_{2} \left( t \right) + \omega_{3} X_{3} \left( t \right)}}{3}$$

where, $\omega_{i} (i = 1,2,3)$ represents respectively the learning rate of wolf $\omega$ to wolf $\alpha ,\beta ,\delta$; $\left\| {X_{i} \left( t \right)} \right\|$ represents the 2-norm of position vector $X_{i} \left( t \right)$, and $X_{\omega } (t + 1)$ represents the position of the offspring gray wolves. The pseudo code of the GNNA is shown as follows (Algorithm 1).

**Figure:**
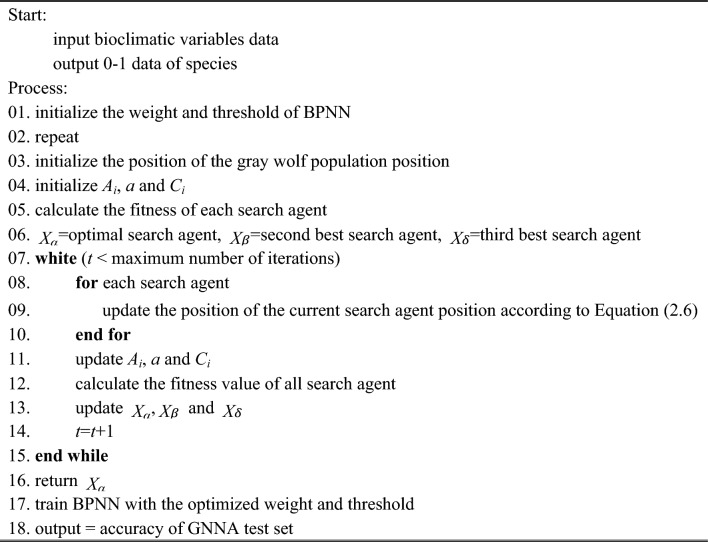
**Algorithm 1** Pseudo code of the GNNA.

### Comparing GNNA predictive performance with BPNN and four commonly used SDMs

We first compared the predictive performance of GNNA with BPNN, posing the explicit hypothesis that GNNA would outperform BPNN and achieve good absolute predictive performance. To this aim, we downloaded occurrence records for 23 species after 1970 from the Global Biodiversity Information Facility (GBIF, http://www.gbif.org/) and removed duplicate records within a 5 km radius. These species have diverse characteristics in the climate, elevation, and range of their habitat (the number of records and details for each species are shown in Table S1 and Table S2 in Supporting Information). We also categorized the 23 species into three kinds of sample sizes according to the number of occurrence records (Table S1 in Supporting Information). In addition, for each species, we randomly generated pseudo-absence data according to three times the number of occurrence records. Each occurrence and pseudo-absence point is associated with a vector composed of climate values, corresponding to bioclimatic variables, which are downloaded from WorldClim 2.1 (http://www.worldclim.org/) at a raw resolution of 2.5 arc-min^51^ and selected by Pearson’s correlation test (r) with |r|< 0.7. Abbreviations and full names of bioclimatic variables are listed in Table S3, and the bioclimatic variables obtained for each species are shown in Table S4.

As a preliminary step, we constructed SDMs for all 23 species through BPNN and GNNA. Specifically, for each species, we first randomly split 80% of the species data into training data and the remaining 20% into testing data. We then evaluated the predictive performance of the model by computing three metrics widely used in ecological research, namely the area under the receiver operating characteristic curve (AUC, Swets^52^), Cohen's kappa (KAPPA, Cohen^53^), and the true skill statistic (TSS, Allouche et al.^54^). We repeated this splitting procedure 12 times and then took the median of the evaluation metrics. In this study, we used a threshold value at which the TSS is maximized to determine presences and absences.

We then applied four commonly used SDMs, namely GLM^14,15^, GBM^16,18^, RF^19^, and MaxEnt^20^, to all 23 species and compared their predictive performance with GNNA. We followed Brun et al.^55^ and Zhang et al.^56^ to set complex parameters for each of the four SDMs involved in the comparison, aiming to make them sufficiently comparable to GNNA. For GLM, the response curve was set to polynomial and the search direction for stepwise regression was set to both; for RF, the number of variables randomly sampled as candidates at each split was set to 5, the number of trees to grow was set to 1000, and the minimum size of terminal nodes was set to 5; for GBM, the maximum depth of each tree was set to 3, the total number of trees was set to 1000, and a shrinkage parameter applied to each tree in the expansion was set to 0.01; for MaxEnt, the maximum number of iterations was set to 100. We performed these SDMs in the R environment (version 4.1.1, R Core Team, 2021) using the packages ‘stats’ (version 4.0.5), ‘randomForest’ (version 4.6–14), ‘gbm’ (version 2.1.8), and ‘dismo’ (version 1.3–5). The data (i.e., species data and bioclimatic variables) and data partitioning used for the four SDMs (i.e., GLM, GBM, RF, and MaxEnt) described above are the same as GNNA and BPNN, which is to facilitate the direct comparison of the predictive performance of the four SDMs with that of GNNA.

### Comparison of spatial distribution predictions—an application case of an invasive species

In addition to the comparison of predictive performance (measured by metrics), the comparison of the prediction of spatial distribution should be taken into consideration. The prediction of spatial distribution is concerned with practical application, especially that of invasive species. We provided an example for predicting the distribution of an invasive plant, *Mimosa bimucronata (DC.) Kuntze* (*M. bimucronata*), which is native in South America and has now invaded the southern coastal region of China. We applied GNNA, BPNN, and the four commonly used SDMs to predict the native and non-native distribution of the species under the current environment, respectively. We used native occurrence records to train the SDMs and predicted both native and non-native potential distributions. At the same time, non-native occurrence records were used to verify the prediction performance of the SDMs for the potential distribution. The occurrence records of *M. bimucronata* in South America were obtained from GBIF (http://www.gbif.org/), and the occurrence records of *M. bimucronata* in China were obtained from the study of Xie et al.^57^. The environmental variables and parameter settings of the SDMs were consistent with those described above in section “Comparing GNNA predictive performance with BPNN and four commonly used SDMs”.

## Results

### Comparison of predictive performance between GNNA and BPNN

**Figure 1:**
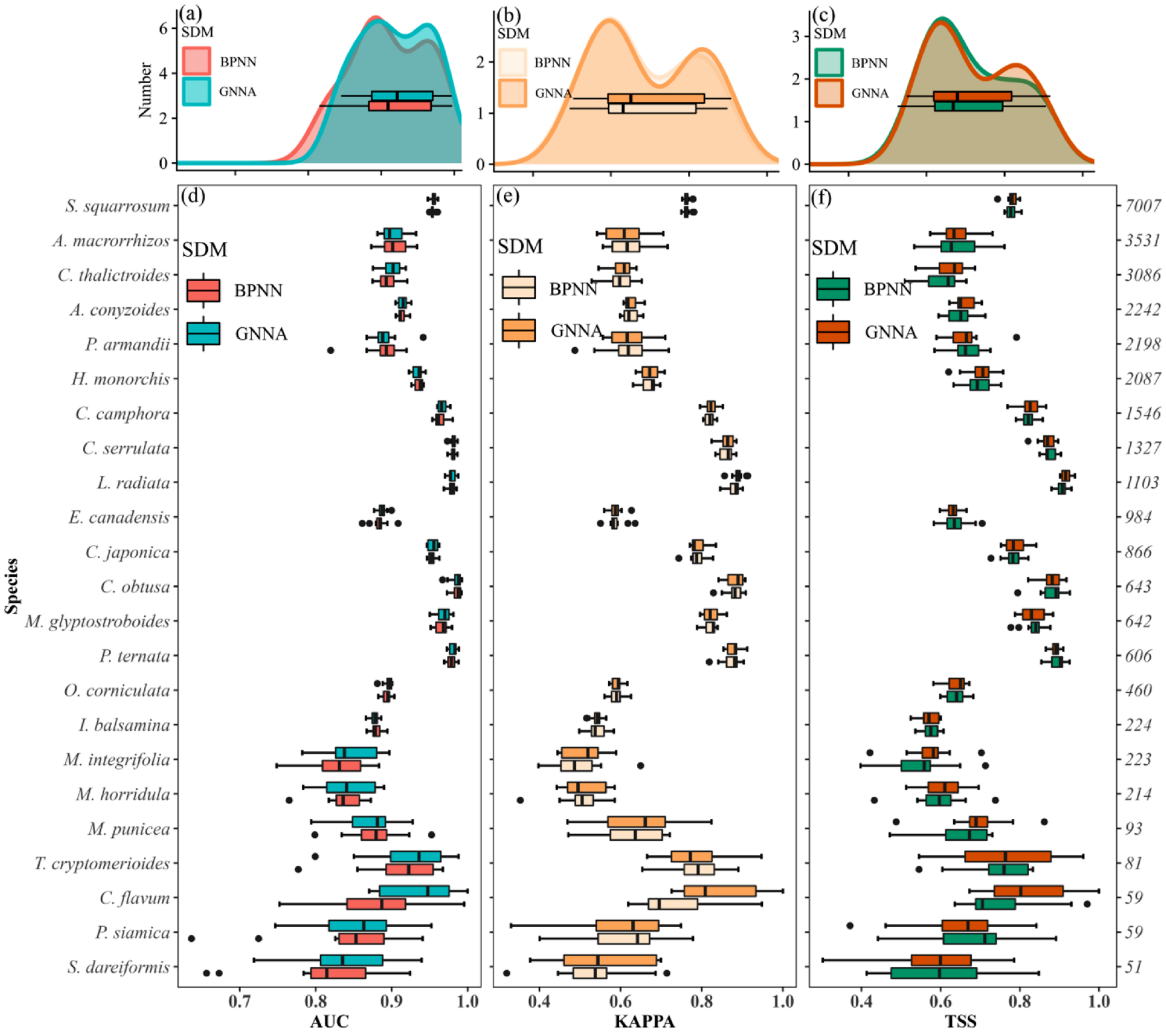
Comparison of predictive performance between GNNA and BPNN under three evaluation metrics (AUC, KAPPA, and TSS). (**a**–**c**) Represent the density distribution of 23 species under AUC, KAPPA, and TSS, respectively. (**d**–**f**) Show the comparison of predictive performance of GNNA and BPNN under AUC, KAPPA, and TSS for each species, respectively.

Overall, the three evaluation metrics consistently showed that GNNA had better predictive performance than BPNN (Fig. 1a–c). Specifically, 20 out of 23 species performed better with GNNA based on having higher metric values for two or more metrics (Fig. 1d–f). The percentage improvement in predictive performance of GNNA over BPNN, no matter which metric was used to measure it, decreased as the sample size increased (Table 1). When the sample size was small, the predictive performance of GNNA was improved by about 2% compared with that of BPNN, while when the sample size was large (middle and big), the predictive performance of GNNA was improved by less than 0.3% compared with that of BPNN (Table 1). The predictive performance of GNNA gradually stabilized with increasing sample size, with a wide inter-quartile range (IQR) when the sample size was small and a narrower IQR when the sample size was large (middle and big) (Table 1).

**Table 1:** Predictive performance of GNNA and BPNN for different sample sizes, measured using AUC, KAPPA, and TSS, percentage improvement in predictive performance (Increment), and inter-quartile range (IQR).

	GNNA			BPNN			Increment			IQR					
	AUC	KAPPA	TSS	AUC	KAPPA	TSS	AUC	KAPPA	TSS	GNNA			BPNN		
										AUC	KAPPA	TSS	AUC	KAPPA	TSS
Small	0.877	0.665	0.667	0.860	0.652	0.653	2.03%	1.99%	2.14%	0.061	0.074	0.065	0.050	0.063	0.062
Middle	0.949	0.834	0.838	0.947	0.833	0.836	0.19%	0.12%	0.24%	0.014	0.019	0.021	0.013	0.021	0.023
Big	0.932	0.688	0.694	0.931	0.687	0.692	0.12%	0.15%	0.29%	0.007	0.009	0.008	0.007	0.015	0.016

### Comparison of predictive performance between GNNA and four commonly used SDMs

**Figure 2:**
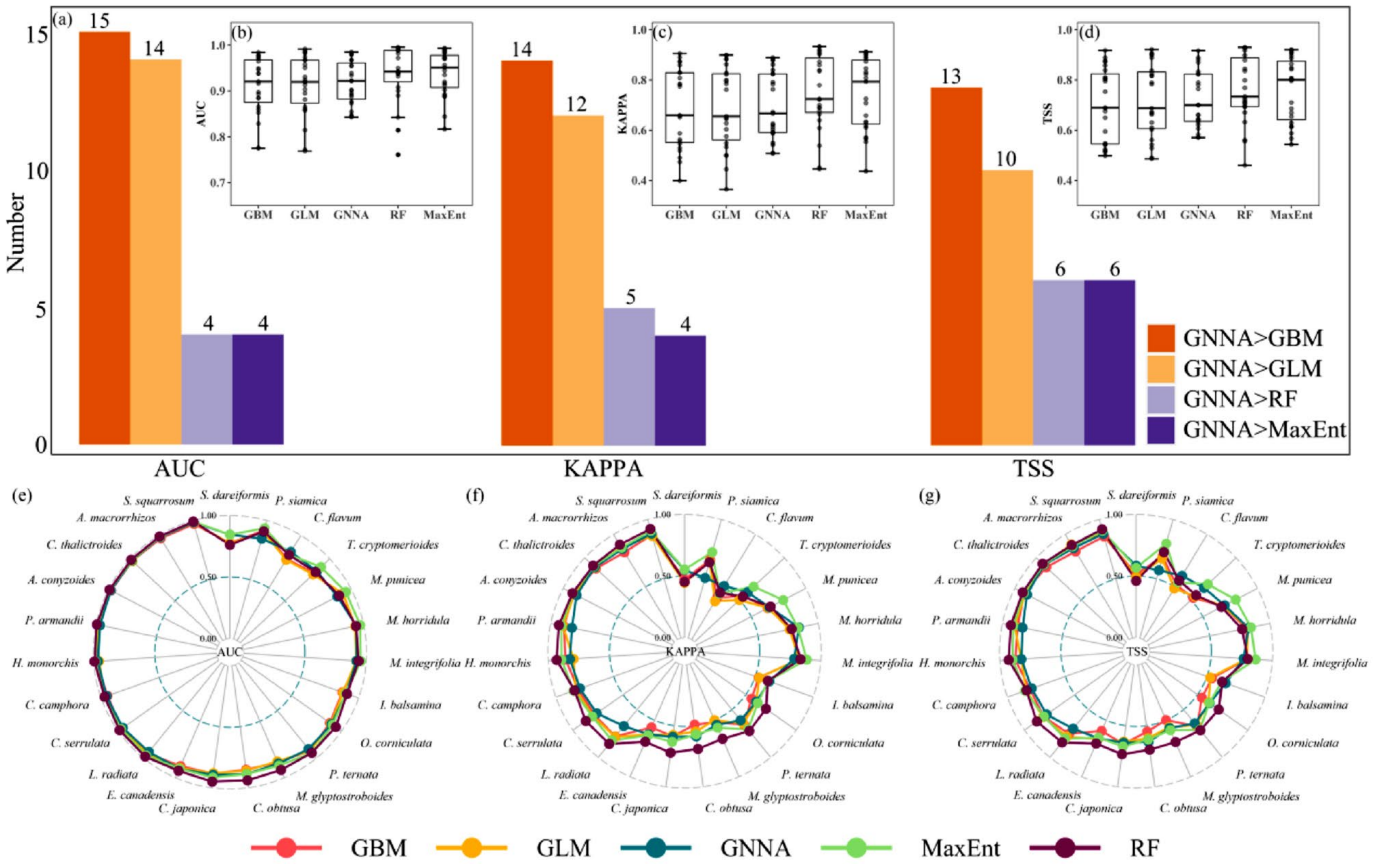
Comparison of the predictive performance of the GNNA model with four commonly used species distribution models (SDMs, i.e., GBM, GLM, RF, and MaxEnt) under three evaluation metrics (i.e., AUC, KAPPA, and TSS). (**a**) Represents how many species out of the 23 species show that GNNA has better predictive performance than four commonly used SDMs. GNNA > ** means that the predictive performance of GNNA is better than that of ** under the same species, and ** means the four commonly used SDMs. (**b**–**d**) Represent the comparison of the predictive performance of GNNA and four commonly used SDMs under the three evaluation metrics, respectively. (**e**–**g**) Represent the predictive performance of the 23 species under GNNA and four commonly used SDMs under the three evaluation metrics, respectively.

Overall, the predictive performance of GNNA was better than that of GBM and GLM, but slightly lower than that of RF and MaxEnt (Fig. 2b–d). Specifically, 14 out of 23 species (about 61% of species) showed better predictive performance of GNNA than GBM, and 12 out of 23 species (about 52% of species) showed better predictive performance of GNNA than GLM (Fig. 2a). Only about five out of 23 species (about 22% of species) showed better predictive performance for GNNA than for RF and MaxEnt (Fig. 2a). The predictive performance of GNNA was comparable to that of MaxEnt and RF in predicting the distributions of species with small sample sizes (such as *S. dareiformis* and *C. flavum*) (Fig. 2e–g).

### Comparison of spatial distributions predicted by GNNA, BPNN, and the four commonly used SDMs

**Figure 3:**
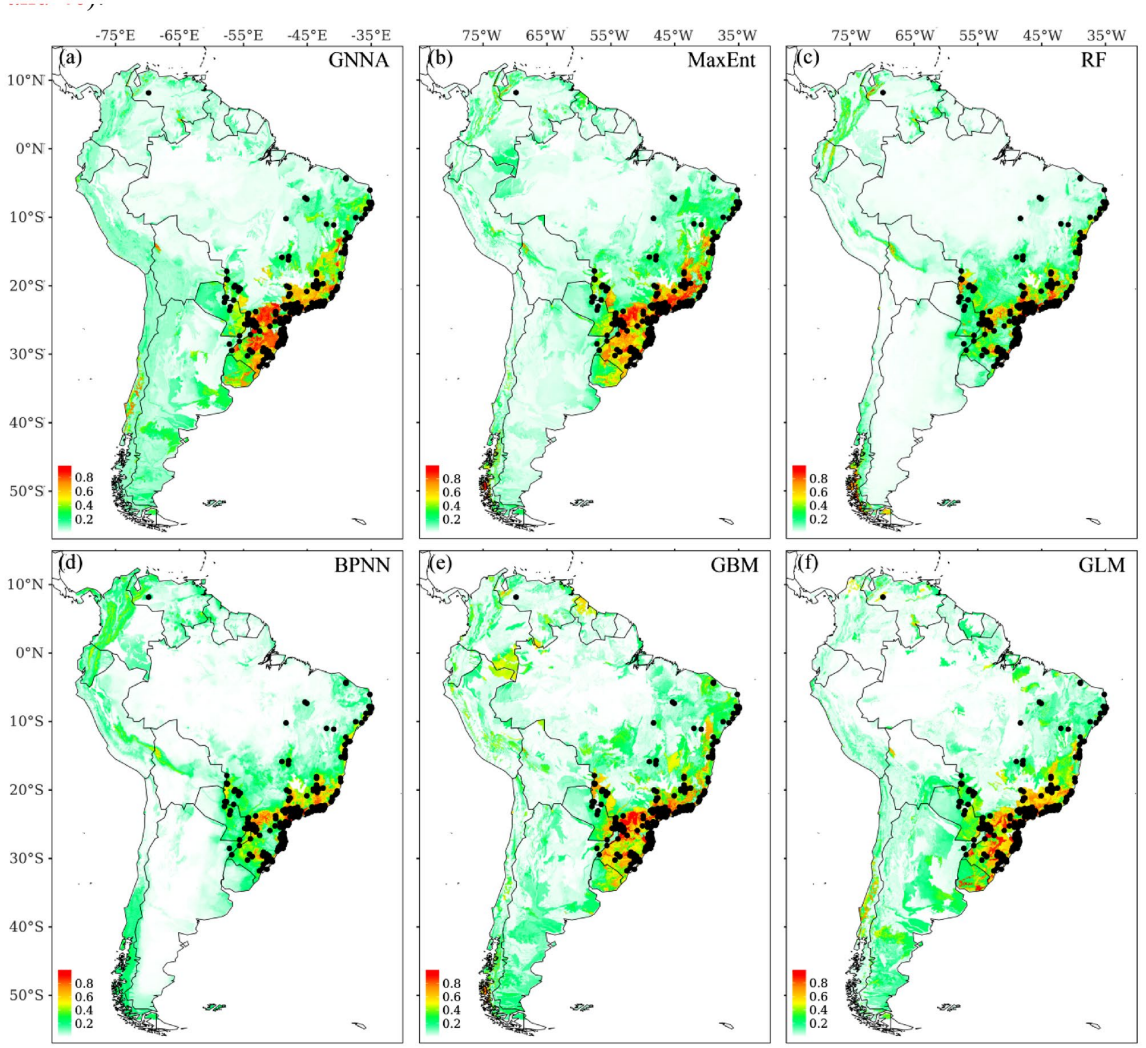
Current distribution of *M. bimucronata* in South America (native) based on GNNA, BPNN, and the four commonly used SDMs (i.e., MaxEnt, RF, GBM, and GLM), respectively. The black point represents the occurrence records of *M. bimucronata* in South America. Figures were created using R 4.1.1 (https://www.R-project.org/).

**Figure 4:**
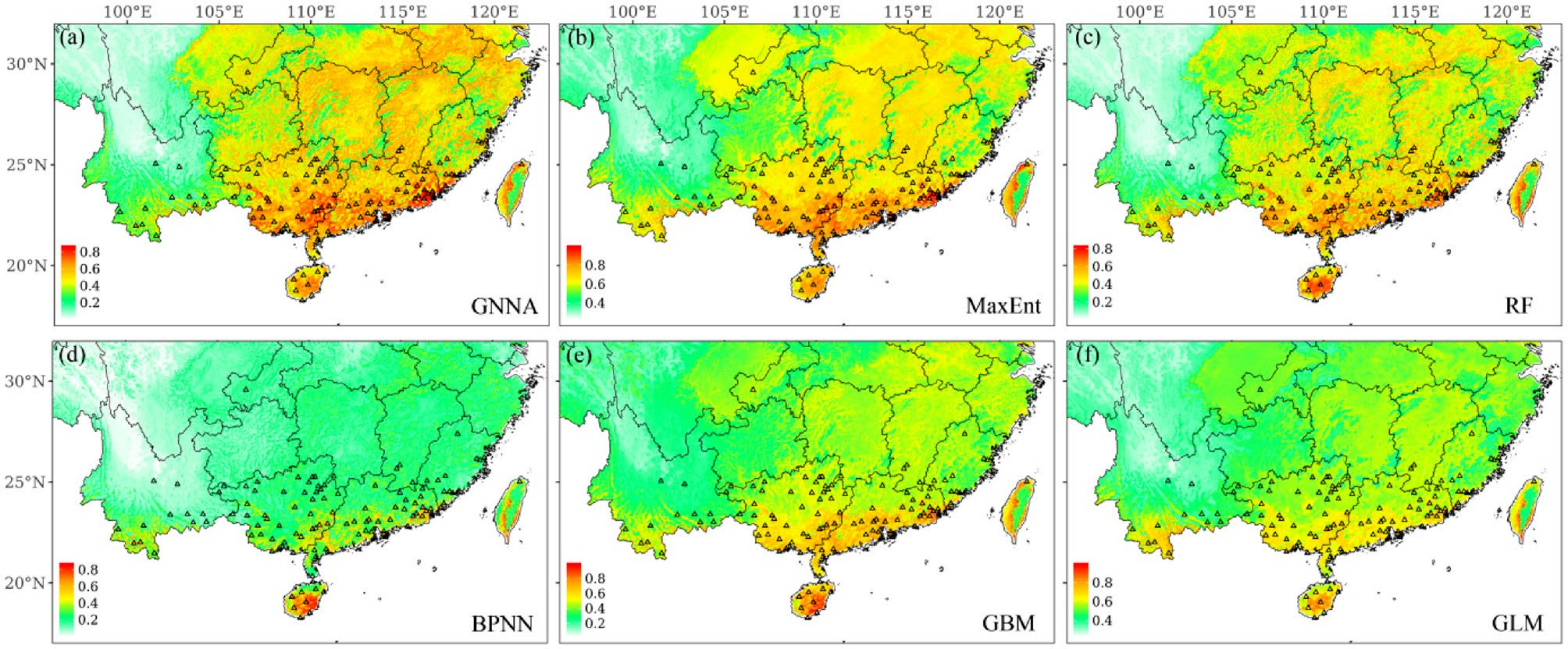
Current distribution of *M. bimucronata* in China (non-native) based on GNNA, BPNN, and the four commonly used SDMs (i.e., MaxEnt, RF, GBM, and GLM), respectively. The black triangle represents the occurrence records of *M. bimucronata* in China. The occurrence records of *M. bimucronata* in China are not used to predict its distribution in non-native (China), but only to test whether the predicted distribution is valid. Figures were created using R 4.1.1 (https://www.R-project.org/).

The native distribution is mainly concentrated on the southern edge of Brazil (Fig. 3), as shown by the almost identical findings from GNNA, BPNN, and the four commonly used SDMs (i.e., MaxEnt, RF, GBM, and GLM) in predicting native distribution areas. However, there are some obvious differences when predicting non-native distribution areas. In addition to the prediction results, all models consistently show that Guangxi, Guangdong, and Hainan are the main distribution areas of non-native species (Fig. 4). The prediction results of GNNA, MaxEnt, and RF also showed a high probability of invasion in Chongqing, which is consistent with the occurrence record of *M. bimucronata* found in Chongqing (Fig. 4a–c).

## Discussion

The proposed hybrid algorithm, GNNA, demonstrates a substantial enhancement in predictive performance over the traditional BPNN, as evidenced by three distinct evaluation metrics. The advancement of predictive performance remains a primary goal in developing new methods for creating SDMs^36,58^, and our research provides a new idea for combining existing SDMs with SIOAs to develop SDMs. In addition, the stability of GNNA is affected by the sample size and increases with the increase in sample size. Nevertheless, certain species within our study did not exhibit this trend when applying GNNA, which may be attributed to either their widespread geographical distribution or potential inaccuracies in occurrence records which sourced from the GBIF.

Our comparative analysis reveals that the predictive performance of GNNA was better than that of GLM and GBM, and delivering predictive results on compare with MaxEnt and RF when species with small sample sizes. Despite the notable superiority of GNNA over the four commonly used SDMs in certain cases (e.g., *S. dareiformis* and *C. flavum*), relying solely on a single SDM could result in skewed interpretations within ecological research^3,59^. It is well-established that no single SDM can consistently deliver high predictive performance across diverse species and regions^29,35,60^. In ecological research, researchers often depend on the consistent results of multiple SDMs or ensemble models to fortify the credibility of their findings^2,23,61–63^. Therefore, our proposed GNNA has great potential to serve as an integral base learner within ensemble model constructions.

Furthermore, biological invasion is a global issue that ecologists have been concerned about for decades^64–67^. Effectively predicting the potential distribution of invasive alien plants provides is crucial for developing prevention and control strategies against their spread^68,69^. SDMs have been increasingly used to predict the potential distribution of invasive plants in recent years^11,57,69^. The GNNA proposed in this study also showed superior ability in predicting the non-native potential distribution of invasive plants.

## Conclusions

This study introduces an SIOA GWO into SDMs, and constructs a hybrid algorithm GNNA to improve the predictive performance of SDMs. Specifically, compared with BPNN, the predictive performance of the hybrid algorithm GNNA proposed in this paper is significantly improved. In addition, GNNA, which has excellent predictive performance comparable to common SDMs such as MaxEnt and RF, can be used as a good base learner for ensemble models. Up to now, many different types of SIOAs have been proposed, and these SIOAs have been tested to have superior optimization capabilities. We will try to combine more SIOAs with SDMs in future work.

### Supplementary Information

Supplementary Tables.

## Data Availability

The cleaned occurrence records for the 23 real plant species investigated in this study: Dryad https://datadryad.org/stash/share/XhPyzK093jJB0x3cyH4x0ujpbDTkAgmqBDDUjZcSh3o.
