# Supplementary material for: A novel hybrid model for species distribution prediction using neural networks and Grey Wolf Optimizer algorithm
Source: Sci Rep. 2024 May 20;14:11505. doi: 10.1038/s41598-024-62285-8 (PMC11106298; doi:10.1038/s41598-024-62285-8)
Supplement: Supplementary file 1 — Supplementary Tables. [file 41598_2024_62285_MOESM1_ESM.pdf]

Supplementary Information for

A novel hybrid model for species distribution prediction using neural networks and grey wolf optimizer algorithm

Authors

Hao-Tian Zhang[1], Ting-Ting Yang[1], Wen-Ting Wang[1,]*

Affiliations

[1]School of Mathematics and Computer Science, Northwest Minzu University, Lanzhou, 730030, P.R. China

*Corresponding Author

Email: iamwwt1983@163.com

Table S1 Names, abbreviation, number of occurrence records, and assigned sample size of species considered for 23 plant species. If the number of species occurrence records is less than 100, the species sample size is classified as small. If the number of species occurrence records is greater than 100 and less than 1000, the species sample size is classified as Middle. If the number of species occurrence records is greater than 1000, the species sample size is classified as big.

Names	Abbreviation	Occurrence records	Sample size
Ageratum conyzoides	*A. conyzoides*	2242	Big
Alocasia macrorrhizos	*A. macrorrhizos*	460	Middle
Camellia japonica	*C. japonica*	866	Middle
Ceratopteris thalictroides	*C. thalictroides*	984	Middle
Chamaecyparis obtuse	*C. obtuse*	643	Middle
Cinnamomum camphora	*C. camphora*	1546	Big

Erigeron canadensis	*E. canadensis*	3086	Big
Cypripedium flavum	*C. flavum*	59	Small
Selliguea dareiformis	*S. dareiformis*	51	Small
Herminium monorchis	*H. monorchis*	2087	Big
Impatiens balsamina	*I. balsamina*	2198	Big
Lycoris radiate	*L. radiate*	1103	Big
Meconopsis horridula	*M. horridula*	214	Middle
Meconopsis integrifolia	*M. integrifolia*	223	Middle
Meconopsis punicea	*M. punicea*	93	Small
Metasequoia glyptostroboides	*M. glyptostroboides*	642	Middle
Oxalis corniculata	*O. corniculata*	3531	Big
Passiflora siamica	*P. siamica*	59	Small
Pinellia ternate	*P. ternate*	606	Middle
Pinus armandii	*P. armandii*	224	Middle
Cerasus serrulata	*C. serrulata*	1327	Big
Sphagnum squarrosum	*S. squarrosum*	7007	Big
Taiwania cryptomerioides	*T. cryptomerioides*	81	Small

Table S2 The characteristics of the 23 plants included habitat, range size, climatic zone, altitude, plant type, habit and whether it was a vascular plant, as well as the source and citation of the species occurrence records.

Abbreviation	Habitats	Range size	Climate zone	Altitude	Plant type	Habit	Vascular plant	Sources and citation
A. conyzoides	Terrestrial	Global	Tropical	>3500	Annual herb	Thermophilic	Y	https://doi.org/10.15468/dl.m6nzsr
A. macrorrhizos	Terrestrial	Global	Tropical	<1000	Perennial herb	Ombrophyte and hygrophilo	Y	https://doi.org/10.15468/dl.a2d9n5

C. japonica	Terrestrial	Global	Tropical and subtropical	<800	Shrub	Thermophilic and hygrophilo	Y	https://doi.org/10.15468/dl.aefvfh
C. thalictroides	Aquatic	Global	Tropical and subtropical	<1000	Aquatic plant	Thermophilic and hygrophilo	Y	https://doi.org/10.15468/dl.9mnyp4
C. obtuse	Terrestrial	Continental	Subtropical	1300-2800	Evergreen	Ombrophyte and hygrophilo	Y	https://doi.org/10.15468/dl.fqqutr
C. camphora	Terrestrial	Global	Subtropical	<1500	Evergreen	Thermophilic and hygrophilo	Y	https://doi.org/10.15468/dl.yks4rm
E. canadensis	Terrestrial	Global	Temperate	30-200	Perennial herb	Xerophilous	Y	https://doi.org/10.15468/dl.pnfne8
C. flavum	Terrestrial	Local	Temperate and subtropical	1800-3450	Perennial herb	Hygrophilo	Y	https://doi.org/10.15468/dl.jtdpsa
S. dareiformis	Terrestrial	Local	Tropical and subtropical	1300-2700	Filicophytin	Thermophilic and hygrophilo	Y	https://doi.org/10.15468/dl.xddrgu
H. monorchis	Terrestrial	Continental	Temperate and subtropical	600-4500	Perennial herb	Hygrophilo	Y	https://doi.org/10.15468/dl.jy6u4g
I. balsamina	Terrestrial	Global	Tropical and subtropical	<1000	Annual herb	Photophilous and thermophyte	Y	https://doi.org/10.15468/dl.tsh7kk
L. radiate	Terrestrial	Continental	Subtropical and temperate	<1200	Perennial herb	Thermophilic and hygrophilo	Y	https://doi.org/10.15468/dl.yb3wsm
M. horridula	Terrestrial	Local	Plateau climate	3600-5100	Annual herb	Psychrophi	Y	https://doi.org/10.15468/dl.f6r964
M. integrifolia	Terrestrial	Local	Plateau climate	2700-5100	Annual, biennial or perennial herbs	Psychrophi and hygrophilo	Y	https://doi.org/10.15468/dl.w8ss39
M. punicea	Terrestrial	Local	Plateau climate	2800-4300	Perennial herb	Psychrophi and hygrophilo	Y	https://doi.org/10.15468/dl.zbmv2c
M. glyptostroboides	Terrestrial	Continental	Temperate and	<1500	Deciduous tree	Hygrophilo	Y	https://doi.org/10.15468/dl.x3jnmf

			subtropical					
O. corniculata	Terrestrial	Global	Tropical and subtropical	10-350	Perennial herb	Thermophilic and hygrophilo	Y	https://doi.org/10.15468/dl.nwd57h
P. siamica	Terrestrial	Local	Tropical and subtropical	540-1600	Liane	Thermophilic and hygrophilo	Y	https://doi.org/10.15468/dl.byrbaj
P. ternate	Terrestrial	Continental	Temperate and subtropical	<2500	Perennial herb	Thermophilic and hygrophilo	Y	https://doi.org/10.15468/dl.pxchzg
P. armandii	Terrestrial	Local	Temperate	1500-3000	Evergreen	Thermophilic and hygrophilo	Y	https://doi.org/10.15468/dl.p7t386
C. serrulata	Terrestrial	Continental	Temperate and subtropical	400-800	Deciduous tree	Thermophilic and hygrophilo	Y	https://doi.org/10.15468/dl.92csxe
S. squarrosum	Wetland	Global	Frigid and temperate	<1000	Bryophyta	Hygrophilo	N	https://doi.org/10.15468/dl.axwe83
T. cryptomerioides	Terrestrial	Local	Tropical monsoon	1500-2500	Evergreen	Thermophilic and hygrophilo	Y	https://doi.org/10.15468/dl.ahye78

Table S3 The environmental variables for our analysis were the 19 bioclimatic variables of current climate (representative of 1970–2000) taken from the WorldClim database version 2.1 (http://www.world clim.org/) with an original resolution of 2.5 arc-min. Abbreviations and full names of 19 bioclimatic variables are listed.

Abbreviations	Full names
BIO1	Annual Mean Temperature\℃
BIO2	Mean Diurnal Range (Mean of monthly (max temp – min temp))\℃
BIO3	Isothermality (BIO2/BIO7) (×100)
BIO4	Temperature Seasonality (standard deviation×100)

BIO5	Max Temperature of Warmest Month\℃
BIO6	Min Temperature of Coldest Month\℃
BIO7	Temperature Annual Range (BIO5 – BIO6)\℃
BIO8	Mean Temperature of Wettest Quarter\℃
BIO9	Mean Temperature of Driest Quarter\℃
BIO10	Mean Temperature of Warmest Quarter\℃
BIO11	Mean Temperature of Coldest Quarter\℃
BIO12	Annual Precipitation\mm
BIO13	Precipitation of Wettest Month\mm
BIO14	Precipitation of Driest Month\mm
BIO15	Precipitation Seasonality (Coefficient of Variation)
BIO16	Precipitation of Wettest Quarter\mm
BIO17	Precipitation of Driest Quarter\mm
BIO18	Precipitation of Warmest Quarter\mm
BIO19	Precipitation of Coldest Quarter\mm

Table S4 For each species, we retained for subsequent comparison analyses bioclimatic variables with Pearson's correlation coefficient (PCC) <0.7.

Species	Bioclimatic variables selected by PCC
A. conyzoides	BIO2, BIO7, BIO10, BIO11, BIO15, BIO16, BIO17, BIO18, BIO19
A. macrorrhizos	BIO2, BIO5, BIO7, BIO11, BIO16, BIO17, BIO18, BIO19
C. japonica	BIO2, BIO5, BIO8, BIO11, BIO13, BIO15, BIO17, BIO18, BIO19
C. thalictroides	BIO2, BIO10, BIO11, BIO15, BIO16, BIO17, BIO18, BIO19
C. obtuse	BIO3, BIO5, BIO7, BIO8, BIO11, BIO15, BIO19
C. camphora	BIO2, BIO7, BIO8, BIO10, BIO11, BIO15, BIO19

C. flavum	BIO2, BIO7, BIO8, BIO10, BIO11, BIO15, BIO16, BIO17, BIO18, BIO19
E. canadensis	BIO2, BIO7, BIO11, BIO18, BIO19
S. dareiformis	BIO2, BIO4, BIO7, BIO15, BIO19
H. monorchis	BIO3, BIO8, BIO10, BIO11, BIO15, BIO18
I. balsamina	BIO2, BIO6, BIO11, BIO16, BIO17, BIO18, BIO19
L. radiate	BIO3, BIO7, BIO8, BIO9, BIO10, BIO11, BIO17, BIO19
M. horridula	BIO2, BIO5, BIO7, BIO8, BIO9, BIO11, BIO17, BIO19
M. integrifolia	BIO2, BIO3, BIO7, BIO11, BIO13, BIO15, BIO19
M. punicea	BIO2, BIO3, BIO12, BIO15, BIO19
M. glyptostroboides	BIO3, BIO8, BIO10, BIO11, BIO15, BIO18, BIO19
O. corniculata	BIO2, BIO7, BIO8, BIO9, BIO10, BIO11, BIO15, BIO17, BIO18, BIO19
P. siamica	BIO2, BIO4, BIO7, BIO9, BIO15, BIO19
P. ternate	BIO2, BIO3, BIO8, BIO10, BIO11, BIO15, BIO18, BIO19
P. armandii	BIO2, BIO3, BIO7, BIO11, BIO15
C. serrulata	BIO1, BIO2, BIO3, BIO7, BIO10, BIO11, BIO15, BIO18, BIO19
S. squarrosum	BIO2, BIO8, BIO10, BIO11, BIO14, BIO15, BIO18, BIO19
T. cryptomerioides	BIO3, BIO11, BIO12, BIO18, BIO19
